# Dr. Orson Gregory Dibble

**Published:** 1916-02

**Authors:** 


**Dr. Orson Gregory Dibble,** N. Y. University 1869, died at his home in Pompey, N. Y., Nov. 24, aged 75. He had been Health Officer of the town for 15 years.

